# Increased expression of TNF ligand-related molecule 1A and death receptor 3 in bladder tissues of patients with painful bladder syndrome/interstitial cystitis

**DOI:** 10.3892/etm.2012.778

**Published:** 2012-10-30

**Authors:** ERWEI ZHANG, XUHUI ZHU, SONG HAN, ZHIFENG PENG, WEI WANG, JUNFA LI, YONG YANG

**Affiliations:** 1 Department of Urology, Beijing Chaoyang Hospital, Capital Medical University, Beijing 100020;; 2 Department of Neurobiology and Beijing Institute for Neuroscience, Capital Medical University, Beijing 100069, P.R. China

**Keywords:** painful bladder syndrome/interstitial cystitis, tumor necrosis factor ligand-related molecule 1A, death receptor 3

## Abstract

Members of the tumor necrosis factor (TNF) superfamily have been revealed to be associated with painful bladder syndrome/interstitial cystitis (PBS/IC). TNF ligand-related molecule 1A (TL1A) and its receptor, death receptor 3 (DR3), belong to the TNF superfamily and have been implicated in chronic inflammatory diseases. Bladder biopsies from 8 female patients clinically diagnosed with PBS/IC according to the National Institute for Diabetes and Digestive and Kidney Diseases criteria and 8 female bladder carcinoma control patients were investigated to test the protein and mRNA expression levels of TL1A and DR3 using western blotting and real-time RT-PCR. The protein level ratio of TL1A to β-actin (IC, 0.65±0.03 vs. controls, 0.25±0.02, P<0.001) and of its receptor DR3 to β-actin (IC, 0.66±0.06 vs. controls, 0.27±0.02, P<0.001) were observed to be significantly higher in the patients with IC. The real-time RT-PCR ΔCts of TL1A minus GAPDH (IC, 7.60±0.52 vs. controls, 10.08±0.32, P<0.001) and the DR3 minus GAPDH (IC, 6.68±0.60 vs. controls, 8.99±0.61, P=0.017) were observed to be significantly lower in the patients with IC, suggesting that the mRNA levels of TL1A and DR3 were higher in the PBS/IC patients. The protein and mRNA expression of TL1A and DR3 are upregulated in the bladder tissues of PBS/IC patients and may be involved in inflammation and apoptosis in PBS/IC.

## Introduction

Painful bladder syndrome/interstitial cystitis (PBS/IC) is a clinical condition that mainly occurs in females. The prevalence of PBS/IC is approximately 1/1,000 (1,2). The disease presents a variety of symptoms, including urinary frequency, nocturia, pain on bladder filling and supra-pubic pain, which may ultimately limit bladder capacity. Despite investigations in the past few decades, IC remains an unresolved problem with regard to its etiology, mechanisms and clinical management.

A number of studies have demonstrated a link between PBS/IC and inflammation (3–7). Members of the tumor necrosis factor (TNF) superfamily (TNFSF) have been revealed to be associated with PBS/IC (6,8,9). TNF ligand-related molecule 1A [TL1A; also known as vascular endothelial growth inhibitor (VEGI) and TNFSF member 15 (TNFSF15)] is an anti-angiogenic cytokine belonging to the TNFSF. TL1A regulates tumor cell behavior and is involved in chronic inflammatory disease (10–12). These studies collectively indicate that TL1A has a role in PBS/IC.

The present study investigated the expression levels of TL1A and its receptor, death receptor 3 (DR3), in bladder biopsy tissues and revealed a potential link between TL1A, DR3 and PBS/IC.

## Materials and methods

### Patients and tissue samples

A total of 8 patients who fulfilled the National Institute of Diabetes, Digestive and Kidney Diseases (NIDDK) diagnostic criteria (13) for PBS/IC and 8 age-matched hematuric controls undergoing cystoscopy for bladder carcinoma were consecutively enrolled in the present study. All participants were at least 18 years old and were enrolled in accordance with the guidelines of the Institutional Review Board of the Beijing Chaoyang Hospital (Beijing, China) and all subjects provided written informed consent.

Following evaluations of the patients through a detailed medical history analysis, physical examination and voiding diary, each patient underwent urinalysis, urine culture, urine cytology and urinary tract ultrasonography analyses. None of the patients had an intravesical treatment history due to urinary infection and PBS/IC. All medications, including antidepressants, antihistamines and steroidal drugs, were discontinued for at least 48 h prior to hydro-distention therapy. No intravesical malignant lesions were detected during cystoscopic evaluations in which hydro-distension up to a pressure of 80 cm H_2_O was administered for 3 min and all biopsies were obtained using the cold cut technique from an area of the posterior bladder which appeared normal after the bladder was emptied. The bladder mucosa biopsies were obtained from the same sites in the control patients with the exception of carcinomas ≥2 cm and were prepared using the same methods for comparison. A total of 4 sections of bladder tissues which were ∼2×2 mm in size were obtained from each patient. Samples were stored in liquid nitrogen immediately and maintained at −80°C until used.

### Western blot analysis

The frozen samples were rapidly thawed and homogenized at 4°C in Buffer C (50 mM Tris-HCl, pH 7.5, containing 2 mM DTT, 2 mM EDTA, 2 mM EGTA, 50 mM 4-(2-aminoethyl)-benzenesulfonylfluoride hydrochloride, 5 mg/ml each of leupeptin, aprotinin, pepstatin A and chymostatin, 50 mM KF, 50 mM okadaic acid, 5 mM sodium pyrophosphate and 2% SDS) and then sonicated to disrupt the tissues completely. Protein concentrations were measured using a BCA kit (Thermo Scientific, Pittsburgh, PA, USA).

SDS-polyacrylamide gel electrophoresis (PAGE) and western blot analysis were performed according to the laboratory’s standard procedure. Firstly, 40 μg total protein from each sample was loaded onto the corresponding lane of a 10% SDS-PAGE gel. Following electrophoresis and the transfer of proteins to a polyvinylidene difluoride membrane (PVDF; GE Healthcare, Waukesha, WI, USA) at 4°C, the PVDF membrane was blocked with 10% non-fat milk in TTBS (20 mM Tris-HCl, pH 7.5, containing 0.15 M NaCl and 0.05% Tween-20) for 1 h. The blocked membrane was incubated with primary rabbit polyclonal antibody against TL1A or DR3 (1:1,000, Abcam Inc., Cambridge, UK) for 3 h. To confirm the uniform loading of the protein, the same PVDF membrane was reprobed with primary mouse monoclonal antibody against β-actin (Sigma-Aldrich Company, St. Louis, MO, USA) at a 1:2,000 dilution for 1 h. Horseradish peroxidase-conjugated goat anti-rabbit or anti-mouse IgG (ZSGB-BIO Inc., Beijing, China) were used as secondary antibodies at a 1:4,000 dilution for a 1-h incubation. An enhanced chemiluminescence (ECL) kit (Applygen Technologies Inc., Beijing, China) was used to identify the signals in the X-ray film. The order of detection of the target proteins was TL1A, DR3 and then β-actin. With each new round of detection on the same PVDF membrane, stripping buffer, containing 100 mM 2-mercaptoethanol, 2% SDS and 62.5 mM Tris-HCl (pH 6.7), was applied and the PVDF membrane was incubated at 55°C until no signals were identified in the X-ray film, indicating that the previously bound antibodies had been stripped from the membrane.

### Total RNA extraction and real-time quantitative RT-PCR

Bladder tissues were homogenized and total RNA was isolated according to the standard operating procedure of the mirVana™ miRNA Isolation kit (Ambion, Carlsbad, CA, USA). RNA was eluted and stored at −70°C. The total RNA concentration was measured using ultraviolet spectrophotometry at 260 nm (NANO 2000, Thermo Scientific), the purity was determined using the 260/280 A ratio and the quality of the isolated RNA was confirmed using 1% agarose gel electrophoresis.

The reverse transcription reactions were performed using the High Capacity cDNA Reverse Transcription kit (GoTaq^®^ 2-Step RT-qPCR System, Promega, Madison, WI, USA) with poly A primers. Real-time RT-PCR for each sample was performed in triplicate using the Fast Real-time PCR System STRATA Mx3000 (Agilent, Boeblingen, Germany). The Ct values and the qPCR were normalized to the GAPDH housekeeping gene using the 2^−ΔΔCt^ method (14). The primers were synthesized by Taihe Gene, Inc. (Beijing, China; Table I).

### Statistical analysis

The experimental data are expressed as the mean ± standard deviation (±s). Statistical analyses were performed using the t-test. P≤0.05 was considered to indicate a statistically significant difference. Statistical analyses were performed using SPSS version 17.0 (SPSS, Inc., Chicago, IL, USA).

## Results

### Description of patients

The clinical characteristics of the 8 IC and 8 control patients are shown in Table II. All patients were female and all subjects who had suffered from an acute bacterial infection within 3 months were excluded. None of the control patients exhibited any symptoms related to IC and biopsy samples were obtained ≥2 cm from the bladder tumor margins. All but 1 of the subjects were postmenopausal. No common medical history was identified among the patients.

### Protein levels of TL1A and DR3

**Figure 1:**
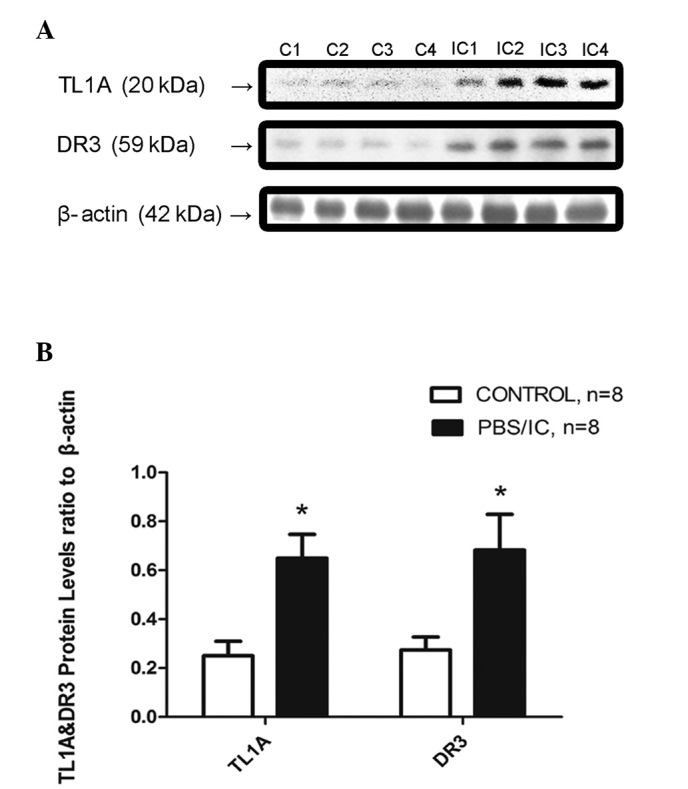
Protein expression differences between TL1A and DR3 in the IC patients and controls. (A) Western blot bands show TL1A and DR3 protein expression levels in the patients and controls. (B) Quantitative analysis shows TL1A and DR3 protein levels in the IC patients to be higher than in the controls. PBS/IC, painful bladder syndrome/interstitial cystitis; TL1A, TNF ligand-related molecule 1A; TNF, tumor necrosis factor; DR3, death receptor 3. ^*^P<0.05 vs. the control group.

The TL1A and DR3 protein expression levels were observed to be increased in the bladder biopsies of the patients with IC compared with those of the controls using western blotting. The TL1A to β-actin ratio in the IC patients was 0.65±0.03 and that in the control group was 0.25±0.02 which was statistically significantly different (P<0.001). The TL1A protein levels of the IC patients were 2.6 fold higher than those of the controls. The DR3 to β-actin ratio in the IC patients was 0.66±0.06 and that in the controls was 0.27±0.02 which was statistically significantly different (P<0.001). The DR3 protein levels of the IC patients were 2.44 fold higher than those of the controls (Fig. 1).

### mRNA expression levels of TL1A and DR3

**Figure 2:**
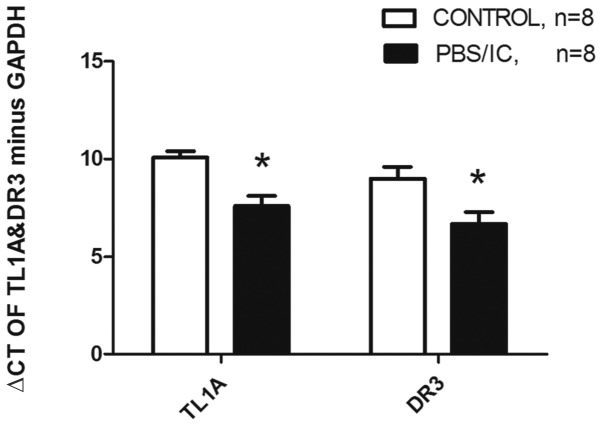
TL1A and DR3 mRNA expression differences between IC patients and controls using the 2^−ΔΔCt^ method. Quantitative analysis shows the ΔCt of TL1A and DR3 minus GAPDH to be lower in the IC patients than in the controls, indicating that mRNA levels of the IC patients are higher than those of the controls. PBS/IC, painful bladder syndrome/interstitial cystitis; TL1A, TNF ligand-related molecule 1A; TNF, tumor necrosis factor; DR3, death receptor 3. ^*^P<0.05 vs. the control group.

The TL1A and DR3 mRNA expression levels were also evaluated in the bladder biopsies using real-time RT-PCR. The ΔCts of TL1A minus GAPDH in the patient and control groups were 7.60±0.52 and 10.08±0.32, respectively, and were statistically significantly different (P<0.001). The TL1A mRNA levels of the patients were upregulated 5.28-fold. The ΔCts of DR3 minus GAPDH in the patient and control groups were 6.68±0.60 and 8.99±0.61, respectively, which was statistically significantly different (P=0.017). The DR3 mRNA levels of the patients were upregulated 4.92-fold (Fig. 2).

## Discussion

PBS/IC is a clinical condition that manifests mainly as storage period symptoms, including frequency and supra-pubic pain, suggesting a mainly sensation-based problem. In the present study, patients with PBS/IC were revealed to have elevated levels of the proteins and mRNA of TL1A and DR3. TL1A is a cytokine belonging to the TNFSF. Three isoforms of TNFSF15 have been reported; TL1A is the most predominant of these isoforms and may be active in inflammation and apoptosis. TL1A has been demonstrated to be significant in tumor cell behavior and chronic inflammatory disease (10,11,15). DR3 is the receptor for TL1A and tumor necrosis factor-like weak inducer of apoptosis (TWEAK). After binding to its ligand, DR3 binds to the adaptor molecule TNF-related apoptosis death domain (TRADD) through its cytoplasmic death domain. TRADD recruitment causes downsteam molecules to activate NF-κB and mitogen-activated protein kinase (MAPK) signaling or triggers caspase activation and programmed cell death under certain conditions (16).

Although the results of the present study suggest an upregulation of TL1A and DR3 in PBS/IC patients, their precise role in this disease was not addressed. However, the known functions of TL1A indicate that it is likely to be involved in the pathogenesis of PBS/IC. Firstly, TL1A is active in inflammation and apoptosis (17–19). The activation of NF-κB in the bladder biopsies of PBS/IC patients (predominantly in the cells of the urothelium and submucosal layer) and apoptosis of endothelial cells in this condition have been reported (20–23). There is also evidence that apoptosis in PBS/IC is mediated by inflammation (23). Upregulation of TL1A and its receptor may trigger inflammation and apoptosis in the bladder, in particular in the elderly population (24). With regard to the evidence that TNF-related apoptosis-inducing ligand and TNFSF14 are also important in PBS/IC (6,8), it may be suggested that TL1A and DR3 elevation is one of the factors contributing to the inflammation and apoptosis in PBS/IC. Secondly, TL1A has been shown to have an anti-angiogenic function (25,26). Upregulation of TL1A and DR3 may inhibit angiogenesis which then causes bladder ischemia and destroys the blood-urine barrier. Previous studies have demonstrated that there is ischemia in PBS/IC patient bladders (27) and impairment of the bladder surface in the PBS/IC condition (22,28). The clinical identification of glomerular bleeding is the only non-exclusive diagnostic evidence of PBS/IC according to the NIDDK criteria. The underlying cause of glomerular bleeding may be the abnormality of the blood vessels arising as a result of this mechanism. Steroid hormone treatments are capable of alleviating the symptoms, which provides supporting evidence for this hypothesis since these drugs inhibit inflammation (29).

The present study has limitations, primarily due to the limited number of patients. The mechanism should be further tested in *in vivo* models. However, there are no widely recognized animal models in which to test this hypothesis. At present, we aim to conduct a larger scale clinical investigation and possibly to specifically block this signaling pathway in a suitable IC disease model, in order to elucidate the precise roles of TL1A and DR3 in PBS/IC. The promising results of the present study justify a larger population study and further mechanistic studies to identify the precise pathogenic role of TL1A and DR3 in PBS/IC and to explore their potential as a possible therapeutic approach.

In conclusion, the present study revealed that TL1A and its receptor DR3 were upregulated in patients with PBS/IC. Therefore, TL1A, a cytokine which triggers NF-κB activation, induces apoptosis and inhibits vascular formation via its receptor DR3, may be important in the pathogenesis of PBS/IC.

## Figures and Tables

**Table I:** PCR primer sequences.

Primers	Sequences (5′-3′)	Product length (bp)
GAPDH		325
Forward	GGCGATGCTGGCGCTGAGTA	
Reverse	ACAGTTTCCCGGAGGGGCCA	
TL1A		145
Forward	CAAACAAGCCAGACTCCATCACT	
Reverse	GAGAACATGGCTCCGAGGTAGAT	
DR3		66
Forward	TGCCGCCGAGACAGCCCCACGAC	
Reverse	GACGGCACGCTCACACTCCTCAG	

TL1A, TNF ligand-related molecule 1A; TNF, tumor necrosis factor; DR3, death receptor 3.

**Table II:** Clinical characteristics of the patients and controls.

Characteristic	PBS/IC	Control
Median age (range, years)	57 (39–78)	59 (42–75)
Gender (female/male)	8/0	8/0
Type of bladder procedure	Hydro-distention	Cystoscopy
Anesthesia	Intravenous	Topical
No. ulcer	0	0
No. glomerular bleeding	8	0
Urinalysis (WBC+-++++)	0	0
Urine culture (+)	0	0

PBS/IC, painful bladder syndrome/interstitial cystitis; WBC, white blood cells.
